# Gene expression profile data for mouse facial development

**DOI:** 10.1016/j.dib.2017.05.003

**Published:** 2017-05-06

**Authors:** Sonia M. Leach, Weiguo Feng, Trevor Williams

**Affiliations:** a Department of Biomedical Research, Center for Genes, Environment and Health, National Jewish Health, 1400 Jackson Street, Denver, CO 80206, USA; b Department of Craniofacial Biology, University of Colorado School of Dental Medicine, 12801 E 17th Avenue, Aurora, CO 80045, USA; c Department of Cell and Developmental Biology, University of Colorado School of Medicine, 12801 E 17th Avenue, Aurora, CO 80045, USA

**Keywords:** Transcriptome, Functional genomics, Ectodermal, Mesenchymal, Embryonic mouse face, Expression profiles

## Abstract

This article contains data related to the research articles "Spatial and Temporal Analysis of Gene Expression during Growth and Fusion of the Mouse Facial Prominences" (Feng et al., 2009) [1] and “Systems Biology of facial development: contributions of ectoderm and mesenchyme” (Hooper et al., 2017 In press) [2]. Embryonic mammalian craniofacial development is a complex process involving the growth, morphogenesis, and fusion of distinct facial prominences into a functional whole. Aberrant gene regulation during this process can lead to severe craniofacial birth defects, including orofacial clefting. As a means to understand the genes involved in facial development, we had previously dissected the embryonic mouse face into distinct prominences: the mandibular, maxillary or nasal between E10.5 and E12.5. The prominences were then processed intact, or separated into ectoderm and mesenchyme layers, prior analysis of RNA expression using microarrays (Feng et al., 2009, Hooper et al., 2017 in press) [1], [2]. Here, individual gene expression profiles have been built from these datasets that illustrate the timing of gene expression in whole prominences or in the separated tissue layers. The data profiles are presented as an indexed and clickable list of the genes each linked to a graphical image of that gene׳s expression profile in the ectoderm, mesenchyme, or intact prominence. These data files will enable investigators to obtain a rapid assessment of the relative expression level of any gene on the array with respect to time, tissue, prominence, and expression trajectory.

**Specifications Table**

**Table:** 

Subject area	*Developmental Biology*
More specific subject area	*Mouse craniofacial development*
Type of data	*html pages*
How data was acquired	*Affymetrix Mouse430v2.0 and Affymetrix MoGene-1.0-st-v1 microarrays*
Data format	*Analyzed*
Experimental factors	*Samples for microarray analysis were pooled microdissected facial prominence as described in*[1], [2]*. Ectoderm was separated from mesenchyme by ‘peeling’ after dispase treatment, as described in Li and Williams**[3]*.
Experimental features	*Gene expression data was compared across age, prominence and tissue layer.*
Data source location	*University of Colorado School of Dental Medicine, Aurora, CO*
Data accessibility	*The raw data (.cel files) and normalized expression data are available at GEO (*www.ncbi.nlm.nih.gov/geo*) under accession numbers*GSE7759*[1]**and*GSE62214*[2]**and at FaceBase (*www.facebase.org*) under accession FB00000803.*
Related research article	[1], [2]

**Value of the data**

• The data is a statistically robust and extensively verified multidimensional gene expression resource for mouse facial development.
• By comparing the transcriptome across age, prominence and tissue layer, the data provide a valuable tool for studying the complex process of craniofacial development.
• The data could contribute to interpretation of gene expression data in mouse mutants.
• The data could contribute to interpretation of clinical genetic data pertaining to facial development, morphology and orofacial cleft pathogenesis.

## 1 Data

**Fig. 1:**
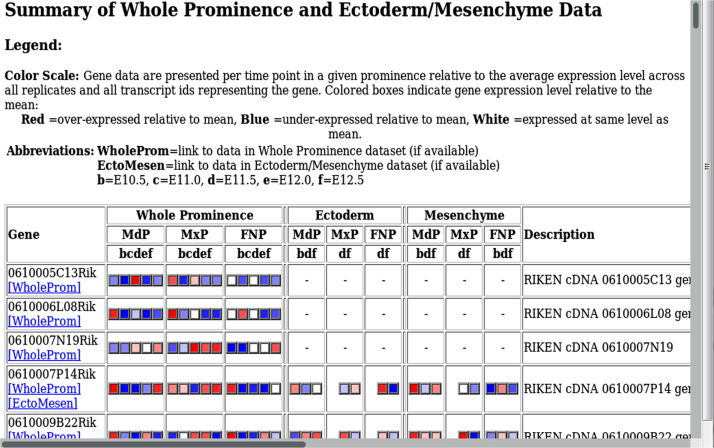
Top-level summary index: An overview of the expression profiles is provided for each named gene in both the whole prominence and ectoderm/mesenchyme datasets, where available. Hyperlinks are provided to access more detailed gene-level data in each dataset separately. The gene list is provided in alphabetical order. Note, as different microarrray chips were used for the two studies, instances where a probeset for a particular gene was available on the microarray for one analysis, but missing for the other, are indicated by a dash. In such instances, hyperlinks in the left column will only be available to connect to that gene in one dataset.

**Fig. 2:**
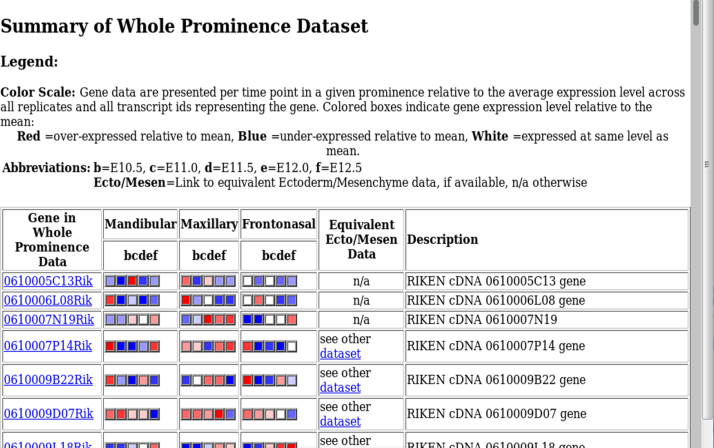
Whole prominence summary index: An overview of the expression profiles in the whole prominence dataset is provided for each named gene, with hyperlinks to the equivalent data in the ectoderm/mesenchyme dataset. If the probesets/gene are not available in the other dataset, this is indicated by n/a.

**Fig. 3:**
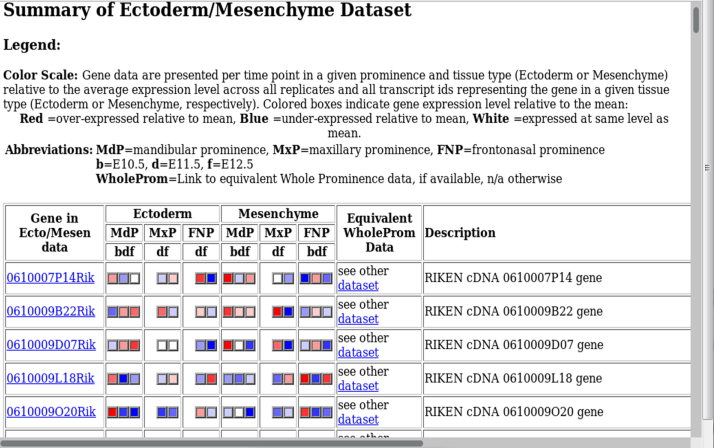
Ectoderm/Mesenchyme summary index: An overview of the expression profiles in the ectoderm/mesenchyme dataset is provided for each named gene, with hyperlinks to the equivalent data in the whole prominence dataset. If the probesets/gene are not available in the other dataset, this is indicated by n/a.

**Fig. 4:**
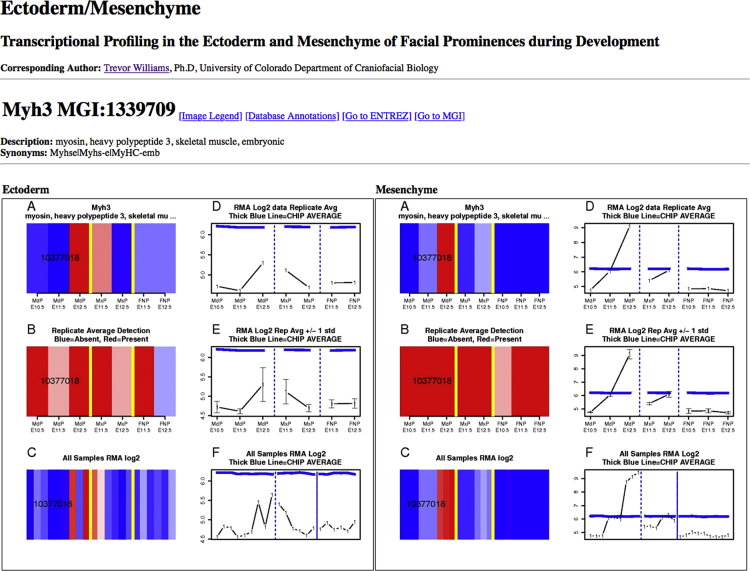
Example profile visualization: Expression signal and detection profiles of all probe sets representing the gene on the microarray are visualized as heatmaps and line graphs (raw and log-scale for whole prominence; log-scale for ectoderm and mesenchyme). Explanatory legend, database annotations, and database references are also available on the html page. Active links also provide access to the legend and the annotations as well as further information on the mouse gene at NCBI and MGI. The gene shown, *Myh3*, has only one probeset in these ectodermal and mesenchymal profiles. Genes with additional probesets will have data from each shown in the equivalent heatmap and as additional line(s) on the line graph.

**Fig. 5:**
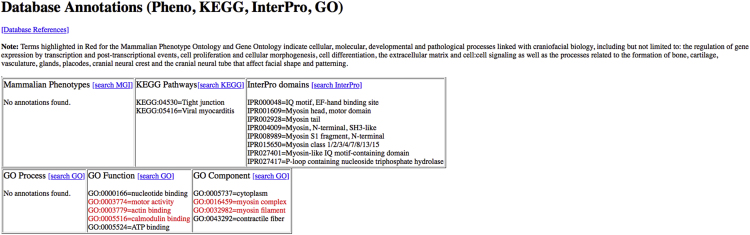
Example Functional Annotation: Database annotations are given for each gene, where terms relevant for craniofacial developmental processes are highlighted in red. The example shown is for *Myh3* (see Fig. 4). (For interpretation of the references to color in this figure legend, the reader is referred to the web version of this article.)

The data are contained in the folder "Trev HTMLs" and within this folder are three items: two folders and an Index.html (see Supplementary material ). The data are designed to be uncompressed in a single location, where hyperlinks use relative file path names to navigate the set of files. Clicking on the top-level Index.html link will provide information concerning the two datasets. This sheet provides an overview, using color-coded boxes for each named gene, to illustrate the gene expression profile found within each of the datasets (Fig. 1). Hyperlinks are also available to access details for each gene in the Whole Prominence or Ectoderm/Mesenchyme datasets. The two folders, WholeProminence and EctoMesen, contain expression profiles and database annotations for every named gene available as.html pages, indexed by gene name for the two studies. Again, within each folder there is a specific index for the whole prominence or ectoderm/mesenchyme dataset (Fig. 2, Fig. 3, respectively). There is also a folder "HTMLS" that connects directly to the list of genes and a folder "JPEGS" which has a list of images used to populate the html pages. Within the whole prominence or ectoderm/mesenchyme datasets, a gene-specific webpage visualizes the expression and detection values for each gene as heatmaps and line graphs (raw and log_2_ scale) (Fig. 4). Each gene-specific webpage also lists annotations from the Mammalian Phenotype [4], Kyoto Encyclopedia of Genes and Genomes (KEGG) [5], InterPro [6] and Gene Ontology (GO) databases [7]. Terms relevant to craniofacial biology are highlighted in red (Fig. 5).

## 2 Experimental design, materials and methods

### 2.1 Data processing

Details of data collection and capture were described previously in [1], [2], [3]. Here, for the whole prominence datasets, the raw image data from the array scans were processed within the Affymetrix GeneChip Operating Software (Affymetrix, Santa Clara, CA) using the option in which a scaling factor was applied to bring the average intensity for all probes on the array to the same target intensity value (TGT) of 500 and exported as Signal.PivotData.dat and Detection.PivotData.dat text files. Transcript identifiers were annotated by gene name, gene description and database identifiers using the Affymetrix-provided annotation files MOE430A.na33.annot.csv and MOE430B.na33.annot.csv. Only data for transcript identifiers associated to a named gene are used for display in the html pages (39103 transcript identifiers corresponding to 22107 distinct gene symbols). All data from our analysis are MIAME compliant and are available via GEO (http://www.ncbi.nlm.nih.gov/geo) with the accession number GSE7759.

For separated ectoderm and mesenchyme datasets, the raw image data from the array scans were processed using the apt-probeset-summarize command from the Affymetrix Power Tools (APT) software suite, with options "-a rma -a pm-only,dabg" to perform 1) Robust Multi-array Average (RMA) with background correction, quantile normalization, and median polish summarization in order to get log_2_ expression signal values, and 2) Detection Above Background (DABG) with unmodified perfect match (PM) intensity values to calculate *p*-values for the probability of reliable signal detection. The DABG values were used to make ׳Absent/Present׳ Detection calls, such that an expression value is called Present if the DABG *p*-value≤0.05, and called Absent otherwise. Transcript identifiers were annotated by gene name, gene description and database identifiers using the Affymetrix-provided annotation file MoGene-1_0-st-v1.na31.mm9.transcript.csv. Only data for transcript identifiers associated to a named gene are used for display in the html pages (25468 transcript identifiers corresponding to 23160 distinct gene symbols). All data from our analysis are MIAME compliant and are available via GEO (http://www.ncbi.nlm.nih.gov/geo) with the accession number GSE62214.

### 2.2 Data visualization and annotation

Data for each gene were visualized using custom scripts in the R language [8]. Functional annotations for each gene were taken from the following databases:

1) **Mammalian Phenotypes**: downloaded 30 Dec 2013 from ftp://ftp.informatics.jax.org/pub/reports/index.html#pheno; see files ftp://ftp.informatics.jax.org/pub/reports/VOC_MammalianPhenotype.rpt and ftp://ftp.informatics.jax.org/pub/reports/MGI_PhenoGenoMP.rpt
2) **KEGG Pathways**: downloaded Feb 2011 from http://www.kegg.jp/kegg/download/)
3) **InterPro Domains**: downloaded 30 Dec 2013 from ftp://ftp.informatics.jax.org/pub/reports/MGI_InterProDomains.rpt
4) **Gene Ontology**: downloaded 30 Dec 2013 from ftp://ftp.informatics.jax.org/pub/reports/index.html#go; see files ftp://ftp.informatics.jax.org/pub/reports/gene_association.mgi and ftp://ftp.informatics.jax.org/pub/reports/go_terms.mgi

## Footnotes

Transparency document Transparency data associated with this article can be found in the online version at doi:10.1016/j.dib.2017.05.003.
